# Three-Dimensional Digital Surgical Planning and Rapid Prototyped Surgical Guides in Bernese Periacetabular Osteotomy

**DOI:** 10.1155/2020/8897066

**Published:** 2020-06-15

**Authors:** Bruno Gonçalves Schröder e Souza, Flavia de Souza Bastos, Valdeci Manoel de Oliveira, Alfredo Chaoubah

**Affiliations:** ^1^Faculdade de Medicina da Universidade Federal de Juiz de Fora, Programa de Pós-Graduação em Saúde Coletiva, Av. Eugênio do Nascimento, s/n, Dom Bosco, Juiz de Fora, MG, Brazil; ^2^Faculdade de Ciências Médicas e da Saúde de Juiz de Fora (SUPREMA), Alameda Salvaterra, 200, Salvaterra, 36033-003 Juiz de Fora, MG, Brazil; ^3^Laboratório de Visualização Científica do Departamento de Engenharia Mecânica da Faculdade de Engenharia da Universidade Federal de Juiz de Fora, Campus Universitário, Rua José Lourenço Kelmer, s/n, São Pedro, 36036-900 Juiz de Fora, MG, Brazil; ^4^Serviço de Ortopedia e Traumatologia, Hospital e Maternidade Terezinha de Jesus, Rua Doutor Dirceu de Andrade, 33, Sao Mateus, 36025-140 Juiz de Fora, MG, Brazil

## Abstract

Bernese periacetabular osteotomy (PAO) developed by Ganz is currently the treatment of choice for skeletally mature symptomatic patients with developmental dysplasia of the hip (DDH) without osteoarthritis. However, the steep learning curve and considerable number of severe complications lead surgeons to seek for alternatives to promote greater reproducibility and safety of this procedure. This is a report of a DDH case surgically treated with the aid of a digital three-dimensional (3D) planning and rapidly prototyped sterile ABS plastic osteotomy guide, developed in Brazil. We present details regarding the planning, guide production, and surgical technique and report the early results of this treatment approach in a single patient. Digital 3D planning and rapidly prototyped surgical guides are applicable and helpful in PAO surgery as shown in this case. We noted no safety issues, good accuracy, and low production costs with this approach.

## 1. Introduction

Bernese periacetabular osteotomy (PAO) as described by Ganz [1] is currently the treatment of choice for skeletally mature symptomatic patients with developmental dysplasia of the hip (DDH) without osteoarthritis [2]. In patients older than 30 years of age, with spherical femoral heads, without advanced hip osteoarthritis, and with good joint congruency, optimal results were reported in up to 60.5% in a 20-year follow-up of the original cohort [3]. In selected cases, disease progression to osteoarthritis was avoided, with good clinical outcomes with up to 30 years of follow-up [2].

Nonetheless, it is a complex and technically demanding surgery, with a steep learning curve [4, 5]. Among the reported PAO surgery complications, intra-articular fractures, posterior column fractures, and damage to vascular structures and nerves are the more severe examples and are directly related to the surgical technique [6, 7].

Rapid prototyping (RP), with 3D-printed surgical guides, and digital 3D surgical planning are increasingly being cited in the literature as useful tools to aid with complex surgeries [8]. In fact, several authors reported potential advantages such as surgical time economy, increased surgical accuracy and precision, and lower complication rates [9]. In 2016, a cadaveric study reported good accuracy and utility of RP-generated guides in simulated PAO [10]. In 2019, the first series of 20 patients undergoing surgery with RP-generated plastic guides reported viability of this approach as well as good accuracy and reproducibility [11].

This study is aimed at reporting an illustrative case of DDH surgically treated with PAO, with the aid of digital 3D surgical planning and sterile RP-generated ABS plastic guides. We provide details on the planning process, strategies to create a low-cost surgical guide, and the early clinical and radiographic outcomes of the patient.

## 2. Case Presentation

A 28-year-old, Brazilian, white female, complaining of right groin pain, presented to the office and reported a previous DDH diagnosis. She had a history of trochanteric pain in her adolescence, but her symptoms worsened and migrated to the inguinal area, and substantially increased in the last year, preventing her from practicing sports or walking long distances. She had sought previous consultation with at least five hip surgeons, who established the diagnosis and indicated surgery, but none of them was able to go on with treatment reporting that they did not feel comfortable with the surgical procedure.

There were no comorbidities, allergies, or medication use, except for analgesics.

On physical examination, there was a mild limp on the right side, no deformities, and some tenderness over the trochanter. Of note, there was an increased range of motion in the internal rotation of the hip and severe pain in the provocative anterior impingement test (in flexion, adduction, and internal rotation).

**Figure 1:**
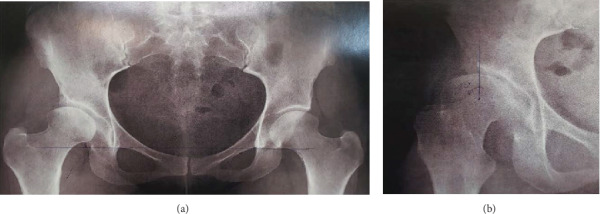
Radiographs of the pelvis (AP in (a) and Lequesne view in (b)) showing signs of hip dysplasia.

Radiographs depicted signs of right DDH, with a Tönnis angle of 20°, a center edge angle of 8°, an anterior center edge angle of 0°, a broken Shenton line, and the presence of a spherical femoral head (Figure 1).

**Figure 2:**
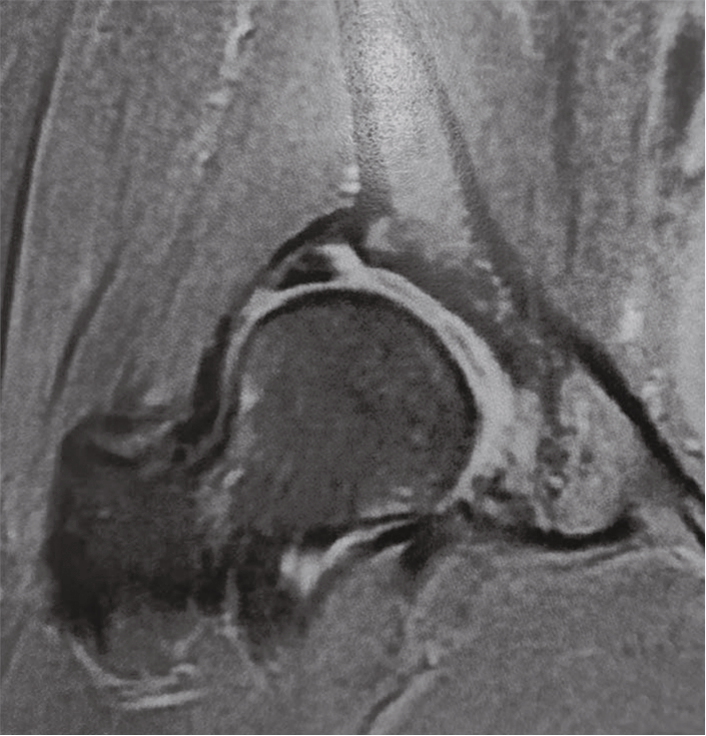
MRI (coronal cut) of the right hip showing dysplasia and a hypertrophic labrum torn at its base.

Computed tomography (CT) scan provided three-dimensional details of the deformity, including the presence of the femoral head-neck junction offset loss, leading to the concomitant cam deformity diagnosis. Magnetic resonance imaging (MRI) contributed to the diagnosis of a typical hyperplasic torn labrum (Figure 2).

Upon confirmation of the diagnosis and clear surgical indication, we suggested to proceed with the treatment. Moreover, we explained that we were in the learning curve for the procedure, although we were aware of the applicability of RP technology as an aid in such situations and had been developing a method since 2016. The patient, who was a dentist, reported her own experience with the great improvements this technology allowed in oral implants and accepted to undergo surgery with the use of a custom-made osteotomy guide generated by RP. After extensive discussion of the potential risks and benefits of the procedure, the patient provided a signed consent for the procedure. This report has been approved for publication by the Ethics Committee, and the patient also provided a signed consent for the publication.

### 2.1. Surgical Planning

CT scan images of the patient's pelvis were imported into the InVesalius 3.0 software as Digital Imaging and Communications in Medicine (DICOM) and converted into surface tessellation language (STL) [12]. The 3D images were manipulated by the surgeon in a CAD/CAM software (Meshmixer, Autodesk), and the femur and the pelvis were split into separate 3D objects.

In sequence, the surgeon simulated a PAO surgery, adding four parallelogram objects created in the software measuring 20 cm long and 3.3 mm thick, in order to represent the surgical osteotomy (width of the solids varied from 15 to 25 mm, depending on the surface it would be used). These objects, named osteotomes, were placed in the pelvis to generate bone cuts according to the original technique [3]. Positioning of the cuts was verified by 3D rotation of the pelvis and by creating plane cuts in three different planes. We saved and named this file as Planned Cuts.

**Figure 3:**
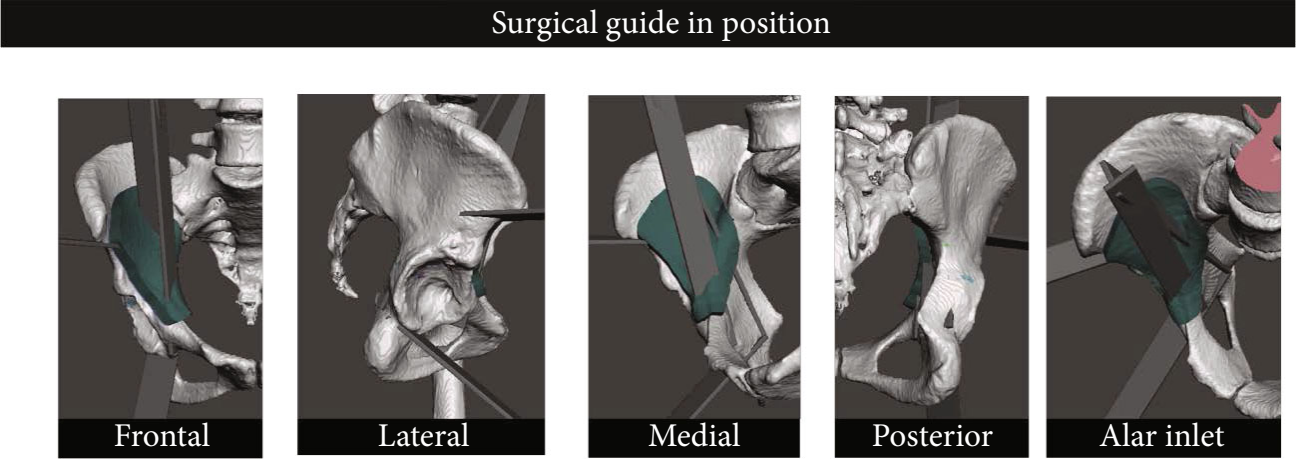
The pelvis with positioned osteotomes and the extruded guide (in green).

After, using the Meshmixer selection tool, the surgeon highlighted the area of the inner pelvis around the entry points of the osteotomes known to be accessible during ilioinguinal approach. The selected area was treated with optimize and smooth boundary tools. An extract tool was then used to create a negative template of the inner pelvis with a 1 mm offset. This selection was then separated to create a new object, which was extruded into a constant direction to produce a 10 mm thick object, named the Guide (Figure 3).

In addition, three slots were created into the guide by Boolean subtraction of the osteotomes. We saved this as an STL file named the Final guide. This custom stereotaxic surgical guide was able to adapt to the inner pelvic surface and guide the surgical chisels during actual surgery after being printed.

**Figure 4:**
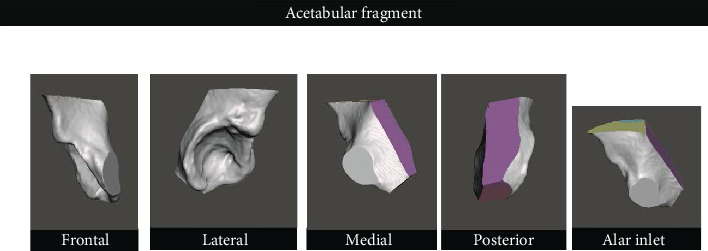
Images of the acetabular fragment in different projections (rotations).

After the guide creation, attention was directed to create a separate acetabular fragment, as in surgery. For that, we used the Planned Cuts file and performed Boolean subtraction operations in which we removed all the osteotomes as well as the other bone fragments, leaving the acetabular fragment. This, too, was saved as a separate object (Figure 4).

**Figure 5:**
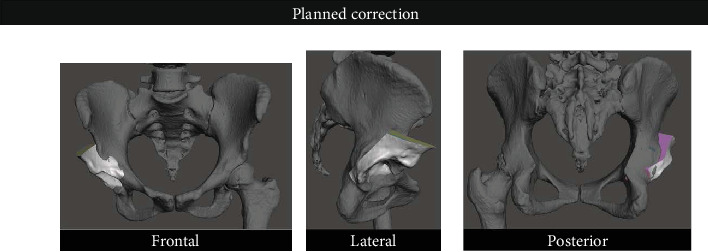
Images of the acetabular fragment superimposed to the pelvis after rotation and space repositioning.

The original file with the separated pelvis and femur was then used, and the acetabular fragment was imported. We used transformation and rotation tools to move the femur into the desired position, in order to restore the Shenton line and the center of rotation to their native site (we used the contralateral healthy hip as a reference). Then, we rotated the acetabular fragment over the center of the femoral head in order to restore normal radiographic parameters (Figure 5).

**Figure 6:**
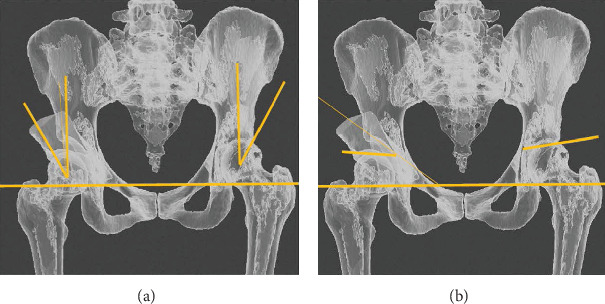
Image of the repositioned acetabular fragment, femur, and pelvis, with the ray casting technique, to simulate the transparency of the structures as in radiographs. The center edge angle (a) and Tönnis angle (b) were measured in both hips.

In order to acquire visualization similar to that in radiographs, the objects were selected and visibility toggled, which produced a transparency effect (ray casting). At the end, we took screenshots of the images and measured the usual radiographic parameters in order to confirm adequate correction and provided the surgeon with visual feedback during surgery (Figure 6).

The surgeon took 32 min to prepare the files to be used in the CAD/CAM software, 21 min to plan the cuts, and 18 min to create the final guide. He took another 1 h and 10 min to create the separate acetabular fragment, rotate and position it according to the plan, and generate a file containing all the images and measurements of the surgical plan. Overall, the process took 2 h and 21 min.

### 2.2. Rapid Prototyping

**Figure 7:**
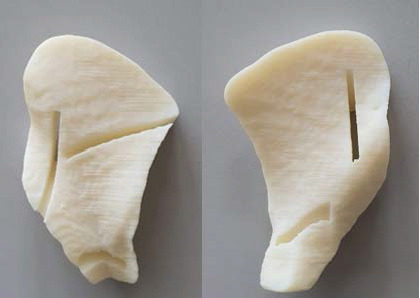
Final guide printed in ABS plastic (pelvic and abdominal faces).

The files containing 3D images of the final surgical guide were exported in STL file format to the 3D printer software (Repetier-Host), and the guides were printed in a commercial-grade fusion deposition material printer (GtMax Core A1, GTMax, Brazil) using a pure, colorless 1.75 mm Acrylonitrile Butadiene Styrene (ABS) plastic (GTMax, Brazil) (Figure 7). After visual inspection for flaws, actual surgical chisels were tested through the slots for compatibility. No problems were observed, and the fit was precise. Printing time was 4 h and 48 min, and 19.06 min was used for the filament (the cost of the filament used was $2.85 BRA).

The hospital sterilized the surgical guide using ethylene oxide according to its routine for plastic materials.

### 2.3. Surgical Technique

Surgery was performed under general anesthesia along with neuroaxis blockade with an epidural catheter. The patient was positioned supine with a small bolster under the right buttock. A bikini-type incision centered 2 cm distal to the anterior superior iliac spine (ASIS) was developed parallel to the skin lines. The external oblique fascia was detached from the iliac crest, and a sponge inserted between the iliopsoas muscle and the iliac wing. The space between the fascia lata tensor and the sartorius muscle was developed with care to protect the lateral femoral cutaneous nerve. The rectus femoris tendon was tagged and divided distal to the confluence of its direct and reflected heads. An osteotomy of the ASIS maintaining the insertion for the external oblique, the inguinal ligament, and the sartorius allowed for its medial retraction. The space between the iliopsoas and the hip capsule was bluntly developed using a Cobb Elevator in a medial and distal direction up to the infracotyloid groove of the ischium, which was confirmed by palpation using long Mayo scissors. With the aid of a blunt retractor, an angled Ganz osteotome was inserted in the space and the osteotome driven into the ischium under fluoroscopic assistance, in order to produce an incomplete osteotomy, leaving the posterior column intact.

**Figure 8:**
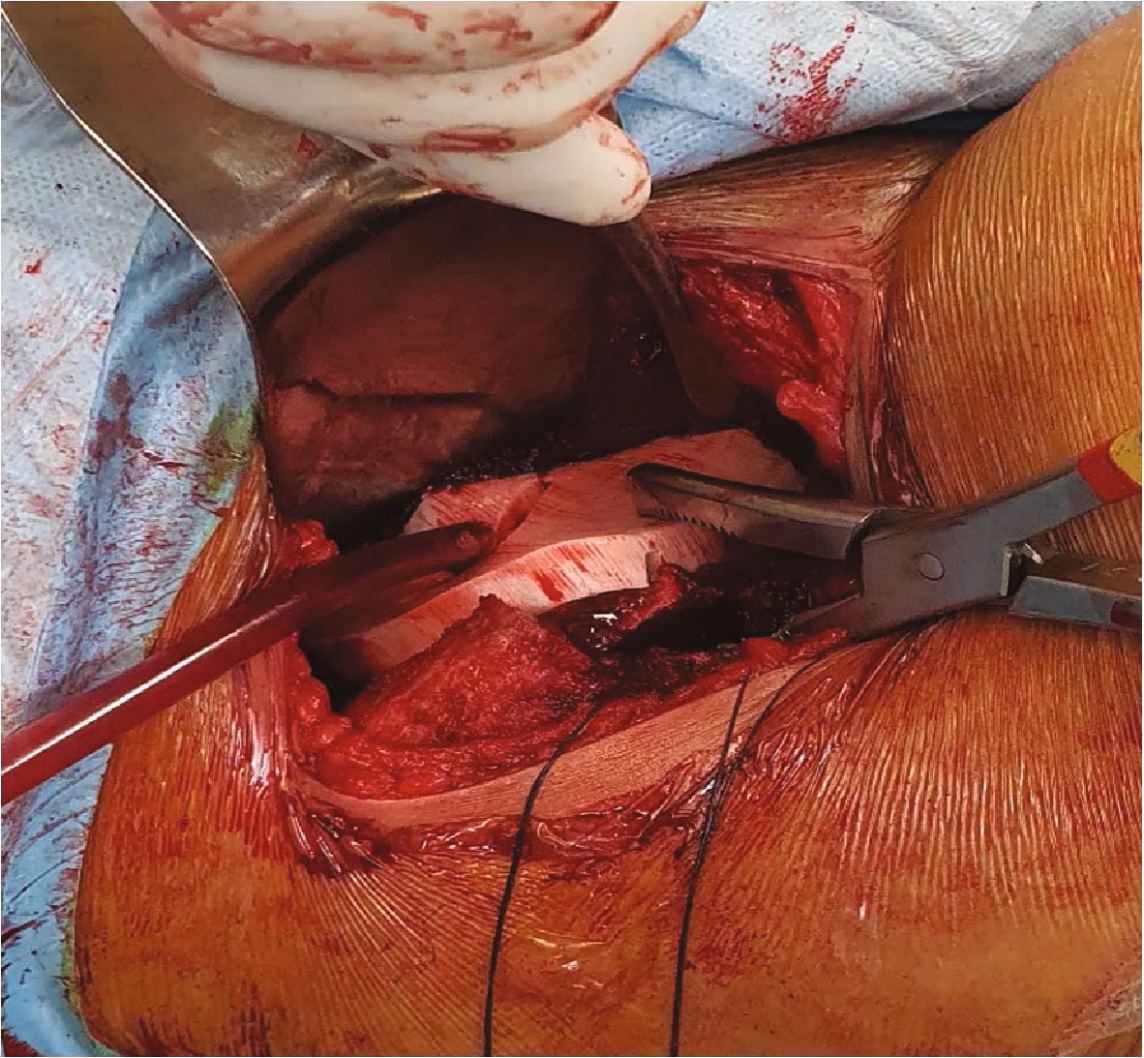
Surgical guide held in place (iliac fossa) with a reduction clamp, and a perfect fit can be noticed.

In sequence, the surgeon exposed the iliopubic ramus medial to the iliopectineal eminence as well as all of the pelvic face of the iliac wing up to the arched line. At this point, the hip was flexed 90° to relax the anterior structures, and the custom-made RP ABS plastic guide was laid on the pelvic surface. Perfect fit was obtained according to plan (Figure 8).

**Figure 9:**
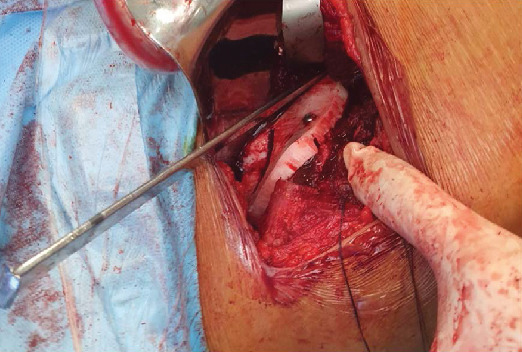
Picture of a straight osteotome inserted into the pubic slot ready to be driven through the bone. Notice the screw securing the guide to the bone.

A surgical reduction clamp held the guide in place while the surgeon attached the guide to the pelvis using a 3.5 mm cortical screw, according to the AO traction screw technique. Optimal congruency between the guide and the bone could be observed. Hence, a straight osteotome (measuring 2.9 × 10 × 200 mm) was introduced into the pubic slot of the guide, and after fluoroscopic confirmation of the position, was hammered into the bone creating the iliopubic bone cut (Figure 9).

**Figure 10:**
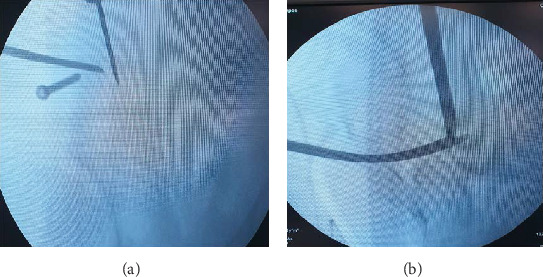
Fluoroscopic images of the iliac and posterior column osteotomes being driven through the bone (a) and the extension of the posterior column cut up to the ischium cut (b).

In sequence, another straight osteotome (2.9 × 20 × 200 mm) was driven through the iliac slot of the guide to produce the osteotomy of that bone up to the slot of the posterior column. That osteotome was then removed. A 2.5 mm drill was used through the posterior column slot to create multiple pivot holes in the thick bone of the posterior column and then replaced by the straight osteotome (2.9 × 20 × 200 mm) creating the posterior column cut. This last cut was developed under fluoroscopy control to verify its depth. The osteotome was inserted down to the incomplete ischium cut (Figure 10).

Of note, the surgeon reported greater confidence and control of the direction of the cuts, and there was less need for fluoroscopy shots. This allowed for a more expedited execution of these steps of the procedure. The guide was removed, and the external part of the posterior column cut was completed using an angled Ganz osteotome through the cut generated by the straight osteotome.

**Figure 11:**
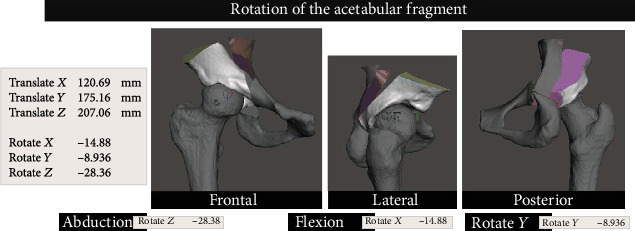
Picture of the acetabular fragment repositioned in space. Note that the planned rotation of the fragment is 5.9° in the longitudinal axis, 28° in the anterior-posterior axis, and 14° in the laterolateral axis.

A 5.0 mm Schanz pin was inserted in the anterior inferior iliac spine, and a reduction clamp grasping the acetabular fragment allowed for its manipulation according to the 3D surgical plan into abduction, flexion, and anteversion (Figure 11).

The position was verified with fluoroscopy, and the fragment was provisionally secured with k-wires. An intraoperative pelvic radiograph was obtained, and the correction was confirmed. The fragment was finally fixed to the pelvis using three 4.5 mm cortical screws. The anterior bone in excess of the acetabular fragment was trimmed away with an oscillating saw. The ASIS was reinserted using a 4.0 mm screw. These steps took 2 h and 30 min.

After that, the hip capsule was opened in a T fashion, the torn hypertrophic labrum was repaired using two absorbable 3.0 mm anchors, and the femoral head-neck junction was reshaped using a burr. The hip was moved in multiple directions, and no impingement was noticed up to 120°, 30°, and 45° of flexion, internal rotation, and external rotation, respectively. The wound was closed by layers, and the skin was closed with a subcuticular nonabsorbable suture. Those additional steps took 50 min, and the total surgical time was 3 h and 20 min.

### 2.4. Postoperative (PO)

**Figure 12:**
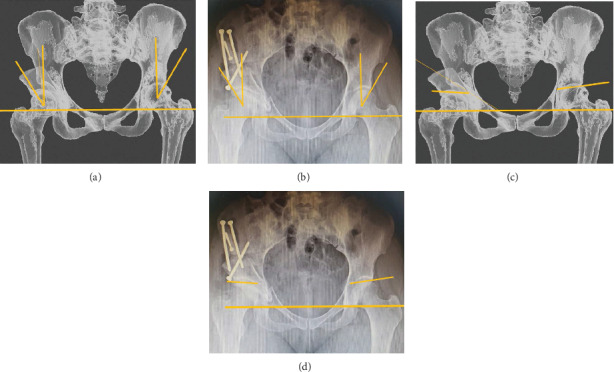
Measurement of the center edge angle (a and b) and the Tönnis angle (c and d), for both the planned (a and c) and actual radiographs (b and d). The obtained center edge angle is equal to as planned, and the Tönnis angle is 2° less than as planned.

The epidural catheter was kept for 24 h. Compression stockinette and antithrombotic prophylaxis with rivaroxaban were then introduced. The patient complained of minimal pain, had no neurological or vascular deficits, and immediate PO hemoglobin level was 10.6 g/dL. On the first day, the patient could sit on the bed without difficulties. Radiographs were taken and showed that the correction was as planned for both the center edge (difference = 0°) and Tönnis angles (difference = 2°) (Figure 12).

On the second day, the PO patient reported minimal pain, with no other complaints. She was able to walk with ease using two crutches, with touch-down weight bearing. She was then discharged with orientations, rivaroxaban for 30 days, and analgesics.

On the 21st day PO, she returned to the office reporting the use of only 10 doses of the prescribed opioid, with no complaints. The sutures were removed, and no signs of complications were observed. Crutches were maintained for 4 months, and circumduction exercises were prescribed. No hip hyperextension was allowed for 6 weeks. At 4 months, the osteotomies had consolidated, and the patient was able to walk without crutches.

## 3. Discussion

Bernese periacetabular osteotomy is an effective surgical treatment for symptomatic adult hip dysplasia [2]. In Brazil, however, its availability is limited to patients due to the lack of surgeons who perform it routinely. Novais et al. [13] suggested that minimum training to perform PAO should be 40 cases. Although the data on Brazilian surgical training is scarce, the fact is that the predicament of our patient, who had access to the largest private insurance in Brazil, illustrates the difficulty of receiving this surgery in Brazil. Although courses, training centers, and mentorship programs are all available for surgeons interested in PAO [14], the steep learning curve and the complications related to the procedure discourage many specialists from executing this procedure.

Rapid prototyping technology has been increasingly cited in medical literature. Specifically in orthopedic surgery, a systematic review reported 38 different applications of RP prior to 2014 [9]. The citations increased over time, especially after 2005. In that study, no report of RP applied to hip preservation surgery was identified. Among the potential benefits of RP surgical guides, one can expect decreased surgical time, greater precision and accuracy, and lower complication rates [9].

We were able to find three different studies reporting the use of RP for periacetabular osteotomies: one experimented in cadavers and the other two in patients. In the first, Otsuki et al. [15] in 2013 developed RP titanium guides for spherical rotational periacetabular osteotomies. Seven patients underwent surgery with the aid of these guides, and the authors reported that no severe complications related to that technique were observed.

In 2016, Zhou et al. [10] performed 10 Bernese PAOs in 5 pairs of cadaveric hemipelvis using a digital surgical plan, RP cutting guides, and RP correction guide. In that experiment, no violation of the articular cartilage or fractures of the posterior column was detected. Good correction of the deformities was reported, and no significant differences between the plan and the actual correction could be observed. In a similar study (unpublished results) with 10 cadaveric hip bones, we obtained comparable results using the guide that we developed and used in this case report.

In 2019, Wang et al. [11] published the first report of the clinical application of RP guides for PAO in 20 patients. The cases managed with this new technique had no complications, and the authors reported good accuracy, less radiation exposure during surgery, and decreased surgical time. This application of RP was reported to be feasible and promising. In our case, such benefits had also been observed as the average surgical time of the surgeon's last cases (4 h and 30 min) has greatly been shortened. Besides using a cutting guide, Wang et al. also produced a correction guide that allowed for three-dimensional positioning of the acetabular fragment. In our case, we only used the 3D virtual plan and the quantitative data derived from that plan to implement the correction during surgery. In that study, the corrections obtained in five different radiographic projections were accurate varying from 1° to 4° on average, which was significantly superior to the traditional method (that varied from 6° to 13°, *p* < 0.01) [11]. In our case, the difference between the planned and actual center edge angle was 0°, and the Tönnis angle was 2°.

The differences between the guides produced by Wang et al. [11] and ours include the printing method, the presence of slots in our guide, and the fact that our guide does not extend to the quadrilateral surface. One potential advantage of our method is that with the slots, the osteotomes are less likely to deviate from course both rotationally and inclination wise, thereby adding accuracy and safety to the procedure. Additionally, as the guide does not extend to the quadrilateral surface, less intrapelvic dissection is needed, which tends to produce less bleeding. In fact, in the study of Wang et al. [11], the hips operated using the newly developed guide bled more compared with the traditional method, on average. However, the authors of that study reported decreased bleeding with experience, and we understand that creating an ischium osteotomy through the window originally described by Ganz [1] in the outer aspect of the pelvis would be less problematic; thus, we did not include this cut in our guide. We observed in our case that there was no need for blood transfusions.

One further difference was the low cost and speed of our method. While we used free software and fusion deposition material (FDM) printing to produce the guides within a few hours, Wang et al. reported taking a whole day at a cost of 1,000 Renminbis (approximately US $140 at the present currency) [11]. Nonetheless, our case illustrates how accurate our guide can be, and the ABS plastic we used was biocompatible, noncarcinogenic, nontoxic, and had been used in other cases with no adverse reactions reported [16]. Potential advantages of this material are its low cost, great resistance to impaction and chemical corrosion, and low fusion temperature that makes its suitable to use with FDM printing. Besides, this material can be sterilized by both gamma radiation or ethylene oxide [17].

Despite formal training in hip preservation surgery, and five previous single surgeries, our case surgeon recounted that the feeling of apprehension persisted due to the known risks and the technical demands of the method. The research process to develop the guide, the actual virtual surgical planning, and the stereotaxic aid of the guide were paramount to overcome the difficulties in this case, which coincide with the experiences reported in the literature [15] [11],. Nevertheless, this study has several limitations, given the nature of a report of a single case, short follow-up, and the customized nature of the guide, which may limit its reproducibility. Albeit it is impossible for us to recommend widespread use of this technique, our initial experience reported in this study was encouraging and future cases will be registered for validation of the results.

## 4. Conclusion

Three-dimensional digital surgical planning and custom stereotaxic rapidly prototyped guides proved to be applicable and useful in Bernese PAO in a Brazilian patient with safety and good accuracy.
